# Intratumoral Injection of *Propionibacterium acnes* Suppresses Malignant Melanoma by Enhancing Th1 Immune Responses

**DOI:** 10.1371/journal.pone.0029020

**Published:** 2011-12-21

**Authors:** Kenshiro Tsuda, Keiichi Yamanaka, Wang Linan, Yoshihiro Miyahara, Tomoko Akeda, Takehisa Nakanishi, Hiroshi Kitagawa, Masato Kakeda, Ichiro Kurokawa, Hiroshi Shiku, Esteban C. Gabazza, Hitoshi Mizutani

**Affiliations:** 1 Department of Dermatology, Graduate School of Medicine, Mie University, Tsu, Mie, Japan; 2 Department of Immuno-gene Therapy, Graduate School of Medicine, Mie University, Tsu, Mie, Japan; 3 Department of Immunology, Graduate School of Medicine, Mie University, Tsu, Mie, Japan; MRC National Institute for Medical Research, United Kingdom

## Abstract

Malignant melanoma (MM) is an aggressive cutaneous malignancy associated with poor prognosis; many putatively therapeutic agents have been administered, but with mostly unsuccessful results. *Propionibacterium acnes (P. acnes)* is an aerotolerant anaerobic gram-positive bacteria that causes acne and inflammation. After being engulfed and processed by phagocytes, *P. acnes* induces a strong Th1-type cytokine immune response by producing cytokines such as IL-12, IFN-γ and TNF-α. The characteristic Th2-mediated allergic response can be counteracted by Th1 cytokines induced by *P. acnes* injection. This inflammatory response induced by *P. acnes* has been suggested to have antitumor activity, but its effect on MM has not been fully evaluated.

We analyzed the anti-tumor activity of *P. acnes* vaccination in a mouse model of MM. Intratumoral administration of *P. acnes* successfully protected the host against melanoma progression *in vivo* by inducing both cutaneous and systemic Th1 type cytokine expression, including TNF-α and IFN-γ, which are associated with subcutaneous granuloma formation. *P. acnes*-treated tumor lesions were infiltrated with TNF-α and IFN-γ positive T cells. In the spleen, TNF-α as well as IFN-γ producing CD8^+^T cells were increased, and interestingly, the number of monocytes was also increased following *P. acnes* administration. These observations suggest that *P. acnes* vaccination induces both systemic and local antitumor responses. In conclusion, this study shows that *P. acnes* vaccination may be a potent therapeutic alternative in MM.

## Introduction

Malignant melanoma (MM) is a life-threatening disease that is commonly resistant to treatment. Early diagnosis followed by surgical resection improves the prognosis of patients with MM. However, despite careful follow-up and treatment with combination chemotherapy or adjuvant therapy, patients with MM frequently develop both local and distant metastases. Patients with distant metastases almost always have a poor clinical outcome. Due to the high frequency of spontaneous recurrence of MM lesions, adjuvant therapy is generally recommended. Starting with Coley toxins more than one hundred ago, adjuvant therapy or immunotherapy has come to be regarded as one of the effective methods for boosting anti-tumor immunity [1], and various compounds and therapeutic modalities have been tested against melanoma using experimental mouse models [2], [3], [4]. However, the precise mechanisms driving the response have not been elucidated.

*Propionibacterium acnes (P. acnes)* is a component of the normal bacterial flora of the skin; it is aerotolerant, anaerobic gram-positive bacteria that plays an important role in the pathogenesis of acne [5]. When dendritic cells (DC) phagocyte and process *P. acnes*, a powerful Th1-type cytokine immune response is elicited, leading to an increased production of IL-12, IFN-γ and TNF-α. Injection of *P. acnes* has been reported to shift a dominant Th2 response to a Th1 type response, with an associated improvement in skin symptoms [6]. Clinically, acne vulgaris, a *P. acnes*-associated disease, often occurs in patients after a recovery from severe atopic dermatitis [7].

In the present study, we developed a mouse model of MM and investigated the clinical and immunological effects of *P. acnes* vaccination.

## Materials and Methods

### Ethics Statement

Animal care was performed according to standard ethical guidelines, and all of the experimental protocols were approved by the Institutional Board Committee for Animal Care and Use of Mie University (Permit Number 21-27-1).

### Mice

Female C57BL/6J(B6) mice were purchased from Japan SLC Co. (Shizuoka, Japan) and were bred under specific-pathogen-free conditions.

### P. acnes

*P. acnes* was purified from normal healthy volunteers and cultured in brain-heart infusion medium supplemented with L-cysteine and Tween-80, as reported previously [8]. Cultured bacteria were washed with sterile distilled water, killed by heating at 60°C for 60 min, and then lyophilized. A strain of *P. acnes* that effectively promotes inflammatory cytokine production was selected (*P. acnes-Mie1*) and used in the subsequent experiments [9].

### Melanoma cell and injection

The B16 melanoma cell line was purchased from ATCC (Manassas, VA), and cultured in RPMI-1640 (Sigma-Aldrich, St. Louis, MO) containing 10% fetal bovine serum (HyClone Laboratories, INC., South Logan, UT) at 37°C in a humidified incubator containing 5% CO2 and 95% air. 1×10^6^ melanoma cells were injected in the dorsal skin of 8 week-old mice.

### Vaccination schedule

3.3 mg of heat-killed *P. acnes* were injected into the dorsal skin at the site where MM cells had been injected in 8–10 week old mice. Mice were divided into five groups: 1) MM-bearing mice treated with PBS (20 µl (MM, n = 14), 2) a group of MM-bearing mice treated with *P. acnes* at 10 weeks old (PMM1, n = 15), 3) another group treated with *P. acnes*-treated at 8 and 10 weeks old (PMM2, n = 15), 4) a control group of mice treated with *P. acnes* (P1, n = 11) at 10 weeks old, and 5) another control group treated with *P. acnes* at 8 and 10 weeks old (P2, n = 13).

### Clinical manifestations and histopathological study

Tumor growth was measured by the longest (L) point and the perpendicular diameter (W) of the dorsal skin tumor mass at 12 weeks of age, and the tumor volume was calculated according to the following formula: 4πW^2^L/3 [10]. The tumor tissue, lung, liver, and spleen were excised from the animals of each group, and samples were embedded in paraffin to prepare sections for haematoxylin & eosin staining.

### Analysis of cytokine mRNA expression

The RNA was extracted from skin lesions using Isogen (Nippon Gene, Tokyo, Japan) according to the manufacturer's instructions and as reported previously [9], [11]. Briefly, 1 ml of homogenate was mixed with 200 µl of chloroform and then centrifuged. The aqueous phase was separated and mixed with 0.5 ml of 2-propanol (Nakalai Tesque, Kyoto, Japan) to precipitate RNA. After centrifugation, the precipitate was washed with 70% ethanol (Nakalai Tesque) and the RNA was suspended in 40 µl RNase-free water. The RNA concentration was measured at 260 nm, and the quality was confirmed by electrophoresis. cDNA was synthesized from 2 µg of RNA using an Archive Kit (ABI, Foster City, CA, USA) according to the manufacturer's protocol. Real time quantitative reverse transcription-polymerase chain reaction (RT-PCR) was performed to determine the transcriptional activity in the tumor lesions. A 25-µl reaction mixture containing 1 µg of cDNA, 900 nmol of each primer, and 250 nmol of TaqMan probe was mixed with 12.5 µl of TaqMan Master Mix (ABI). Quantitative RT-PCR for cytokine transcripts was performed using prequalified primers and probes corresponding to IFN-γ T-bet, IL-12p35, IL-12p40, TNF-α IL-17A, IL-10, MIP-2, and GAPDH (ABI). The ΔδCt method was used to standardize the transcripts to GAPDH.

### Cell isolation and preparation from spleen and skin tumor tissue

The spleens were sampled and single cell suspensions was prepared by mechanical mincing, as reported previously [6]. For the characterization of tumor infiltrating lymphocytes (TIL), skin tumor tissue from the dorsal region was removed, minced gently with scissors and then single cell suspensions were prepared. After passing through a 70-µm-pore mesh, the cells were washed and resuspended with PBS. After Ficoll (SIGMA, St. Louis, MO) separation, cells were washed and resuspended in RPMI1640 medium containing 10% FBS.

### FoxP3 intracellular Staining

The spleen cells were initially stained with FITC-labeled anti-mouse CD4 antibody and PE/Cy5 anti-CD25 antibody, and then fixed in FoxP3 Fix/Perm solution (BioLegend); the cells were then stained with PE conjugated anti-mouse FoxP3 antibody (BioLegend). The fluorescence profile was analyzed by flow cytometry using FACSCalibur (BD Biosciences, San Jose, CA).

### *In vitro* stimulation for cytokine production

Spleen cells were seeded at 2×10^6^ cells/ml (1 ml/well) and cultured in 24-well plates (Costar, NY, USA) in RPMI 1640 medium containing 10% (v/v) FBS (5% of the murine serum was used for IL-10 analysis), 2.0 mM L-glutamine, 100 U/ml penicillin, and 100 µg/ml streptomycin. TIL cells were cultured in 96-well culture plates (Costar) under the same conditions. Cells were stimulated with 1 µg/ml of anti-mouse CD3e (BD Pharmingen, San Jose, CA), 2 µg/ml anti-mouse CD28 (BD Pharmingen) and 1 µg/ml brefeldin A (Biolegend, San Diego, CA). The cells were incubated for 8 h at 37°C under a 5% CO_2_/95% air atmosphere.

### Flow cytometric immunofluorescence analysis

The cell surface antigen and the intracellular cytokines were stained according to the formal Cell Surface Immunofluorescence Staining Protocol and Intracellular Cytokine Staining Protocol (BioLegend), as previously reported [6], [11]. Briefly, for the detection of IFN-γ, IL-17, IL-10 and TNF-α, the cells were first stained with PE anti-mouse CD8 and PE/Cy5 anti-mouse CD3e antibodies (BioLegend). After treatment with the fixation buffer (BioLegend) and the permeabilization wash buffer (BioLegend), cells were stained with FITC-conjugated anti-mouse IFN-γ, IL-17A, IL-10 and TNF-α antibodies (BioLegend). For the characterization of monocytes, the cells were similarly stained with PE-conjugated anti-mouse CD14 monoclonal antibody, followed by intracellular cytokine staining. The fluorescence profiles were analyzed by flow cytometry using FACSCalibur.

### Statistical analyses

Statistical analysis was performed by using the Kruskal-Wallis nonparametric analysis of variance with *post hoc* analysis using the Dunn multiple comparison test. A *P*-value of less than 0.05 was considered to be statistically significant.

## Results

### Cutaneous manifestations ∼Dramatic regression of the tumor by P. acnes treatment∼

**Figure 1:**
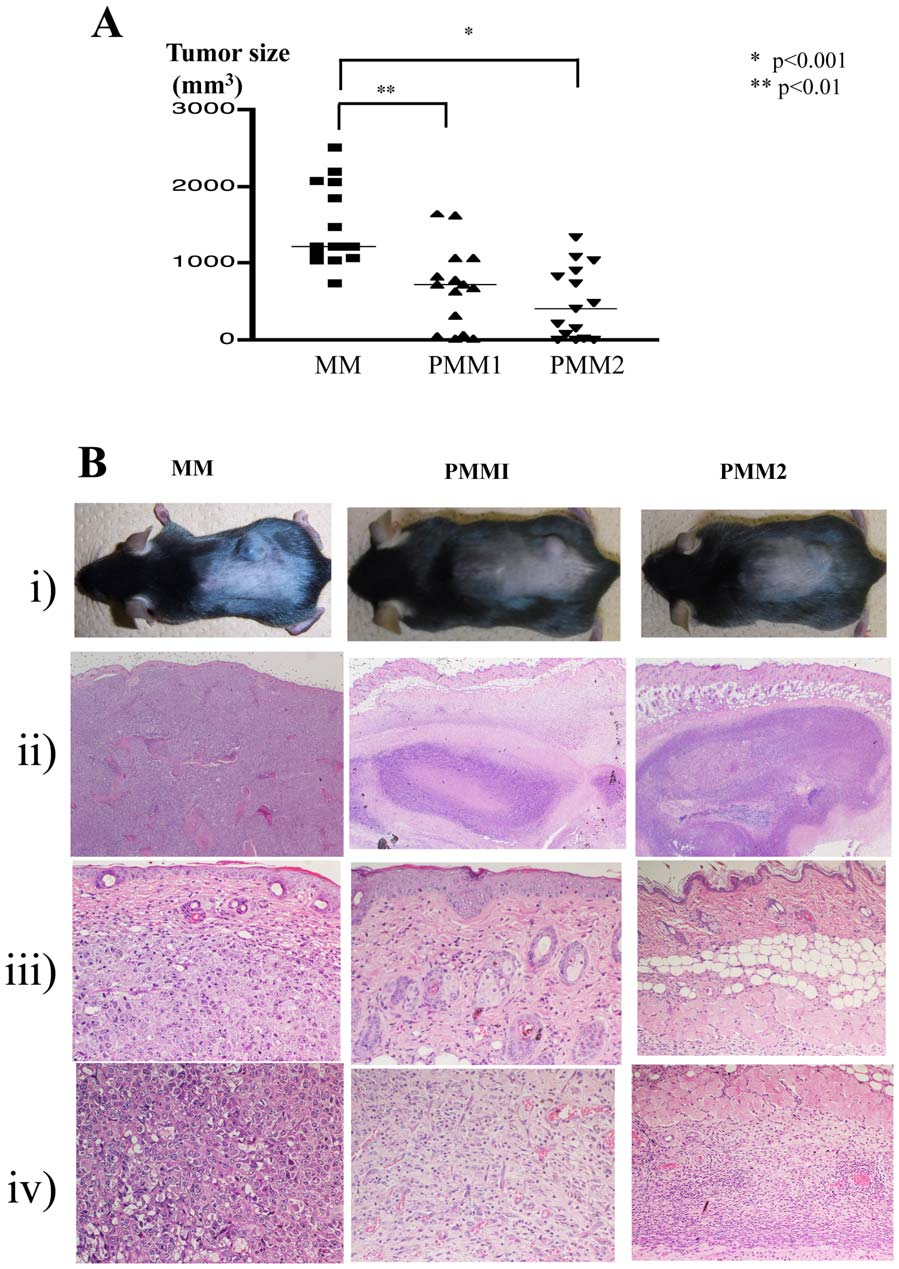
Tumor size, clinical phenotype, and histological findings. **A.** Tumor size at the end of observation period: 4 weeks after B16 melanoma implantation. PBS-treated control mice (MM) developed a large tumor. Treatment with *P. acnes* led to a significant decrease of tumor size in both PMM1 (p<0.01) and PMM2 (p<0.001). **B.** i) Clinical picture of the tumors implanted on the dorsal skin. ii) Massive melanoma cell proliferation in the subcutis with invasion of the surrounding muscular tissues in MM. By contrast, PPM1 and PMM2 displayed subcutaneous granuloma formation (×40). iii) Close up of the upper dermis (×200). iv) Close up of the muscular tissue levels (×200). In MM mice, melanoma cell growth was observed without inflammatory cell infiltration. In PMM1 mice, melanoma cells were detected around the muscular lesion, but melanoma cells were almost undetectable, and abundant mononuclear cell infiltration was found in the PMM2 mice.

We assessed the tumor size 4 weeks after the injection of the B16 melanoma cells. PBS-treated control mice (MM) had developed large tumors. The tumor size in the *P. acnes*-treated melanoma-bearing mice, PMM1 and PMM2, was significantly smaller than in the control mice (Fig. 1A, B).

### Histopathological findings

In the MM control mice, melanoma cells proliferated massively in the subcutaneous region and invaded the underlining muscular layers. Inflammatory cell infiltration was sparsely detected both inside and around the tumors (Fig. 1B). In contrast, histopathological study in PMM1 revealed subcutaneous granuloma formation with only a small number of melanoma cells around the muscular layer. In PMM2, there was enhanced granuloma formation and mononuclear cell infiltration compared with that detected in PMM1. Surprisingly, melanoma cells were almost undetectable in PMM2. No distant melanoma cell metastasis was detected.

### Flow cytometric analysis of spleen cells

**Figure 2:**
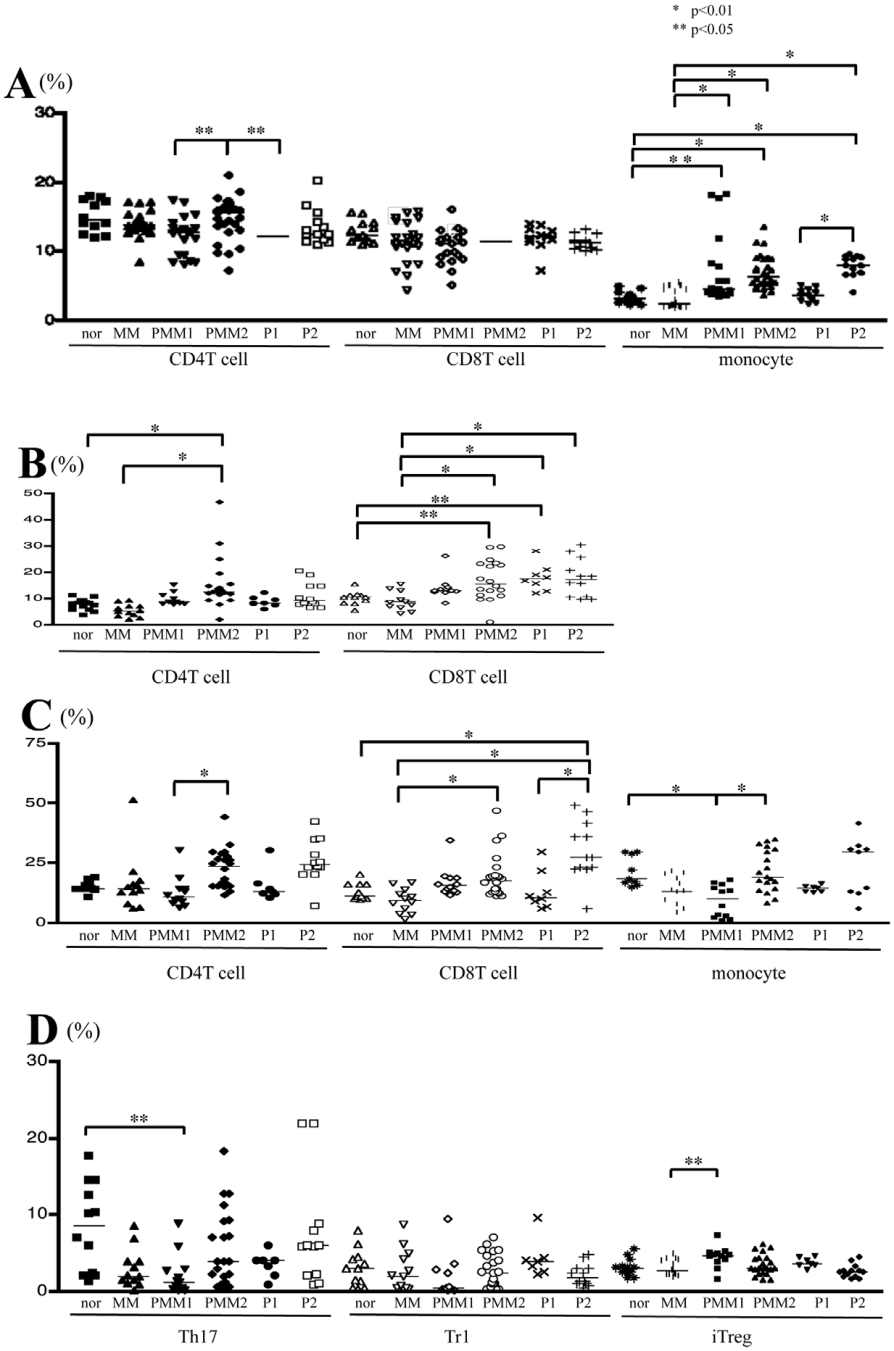
Intracellular cytokine and Foxp3 expression in spleen cells. **A,** Phenotypical analysis: the ratios of the CD4^+^ T cells to total lymphocytes, CD8^+^ T cells to total lymphocytes and CD14^+^ monocytes to total splenic mononuclear cells are shown. The percentage of CD14^+^ monocytes was significantly increased in the PMM2, PMM1, and P2 groups compared to the MM and control groups, respectively. **B,** The percentage of IFN-γ^+^CD4^+^ and IFN-γ^+^CD8^+^ T cells among the total CD4^+^ and CD8^+^ T cells, respectively. In PMM2 mice, the percentage of both populations was significantly elevated compared to the MM and control mice. CD8^+^IFN-γ^+^ T cells were also increased in each of the P1 and P2 groups. **C,** The percentage of TNF-α^+^CD4^+^, TNF-α^+^CD8^+^ T cells and TNF-α^+^ monocytes among the total CD4^+^, CD8^+^ T cells, and monocytes, respectively. In PMM2 mice, the percentage of TNF-α^+^CD8^+^ T cells was significantly elevated compared to MM. TNF-α^+^CD8^+^ T cells were also increased in the P2 group. **D,** The percentages of Th17, Tr1 and iTreg in CD4^+^ T cells. The percentages of IL-17^+^CD4^+^ (Th17) cells and IL-10^+^CD4^+^ (Tr1) cells among the total CD4^+^ cells, and the ratios of the Foxp3^+^CD4^+^CD25^high^ T cells (iTreg) to CD4^+^ T cells, were unchanged by *P. acnes* treatment.

The percentage of CD14^+^ monocytes in the total number of spleen cells was significantly increased in PMM2 and PMM1 compared with the MM and control groups. This increase was also observed in the *P. acnes*-treated control mice (P1 and P2) (Fig. 2A). The percentages of IFN-γ^+^CD4^+^ and IFN-γ^+^CD8^+^ T cells among the total number of CD4^+^ and CD8^+^ T cells were significantly elevated in PMM2 mice compared with MM and control mice (Fig. 2B). CD8^+^IFN-γ^+^ T cells were also increased in the P1 and P2 groups. Similar results were observed in TNF-α expressing CD8^+^ T cells (Fig. 2C). The ratio of IL-17^+^CD4^+^ (Th17) cells and IL-10^+^CD4^+^ (Tr1) cells among the total number of CD4^+^ cells remained unchanged. The ratio of Foxp3^+^CD4^+^CD25^high^ T (iTreg) cells was also not changed by *P. acnes* treatment (Fig. 2D).

### Cytokine mRNA expression in skin lesions

**Figure 3:**
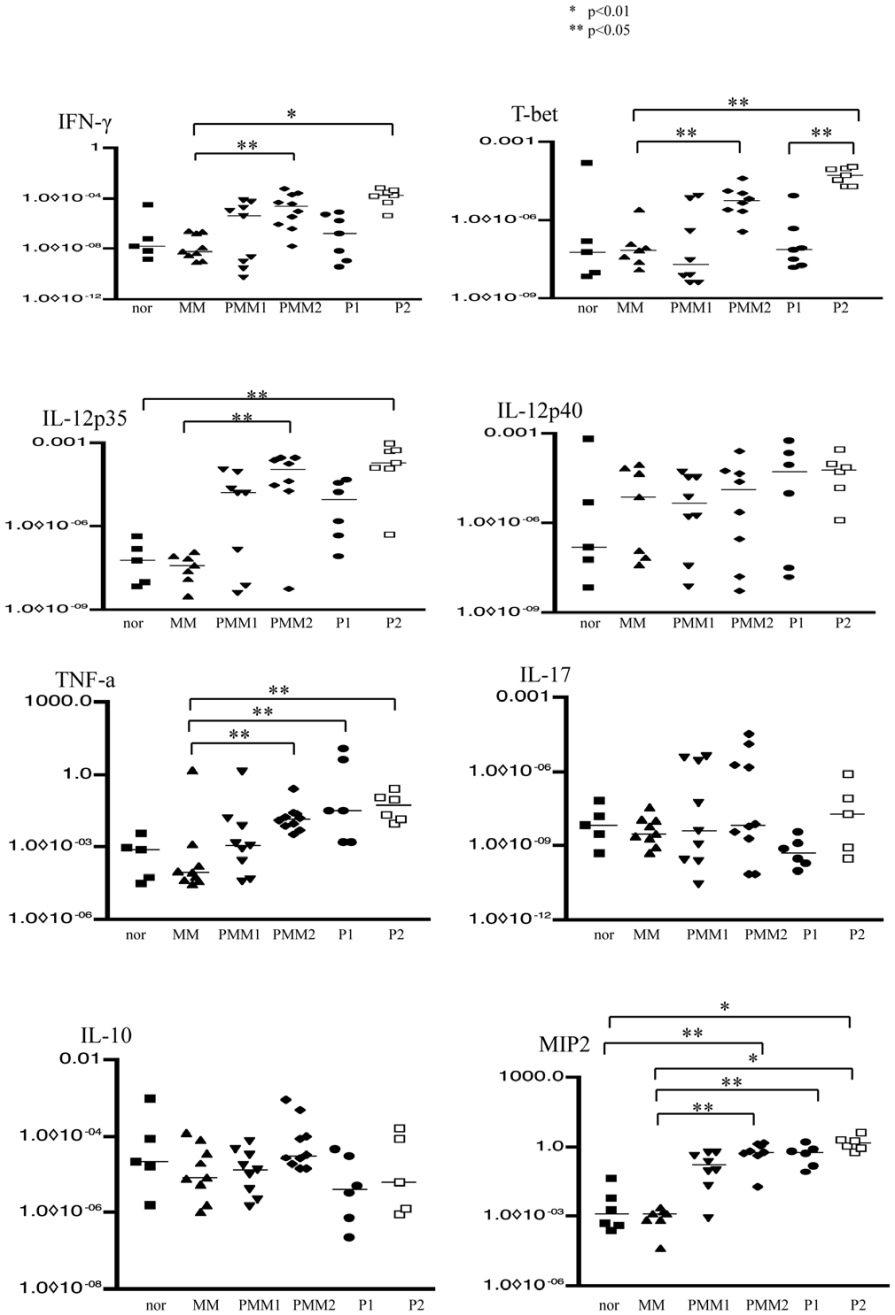
Cytokine mRNA expressions in the skin lesions. Quantitative RT-PCR was performed to determine the cytokine mRNA expression in the tumor lesions. The IFN-γ T-bet, IL-12p35, TNF-α and MIP2 mRNA levels in PMM2 and P2 mice were significantly increased compared to MM mice. No significant change was found in the IL-12p40, IL-17 or IL-10 mRNA levels among the six groups of mice.

Quantitative RT-PCR was performed to investigate the cytokine mRNA expression in the tumor lesions. The RNA expression of IFN-γ T-bet, IL-12p35, TNF-α and MIP2 was significantly increased in PMM2 and P2 mice compared with the MM group. No significant change was found in the mRNA expression of IL-12p40, IL-17 or IL-10 among the six groups (Fig. 3).

### Characterization of tumor infiltrating lymphocytes

**Figure 4:**
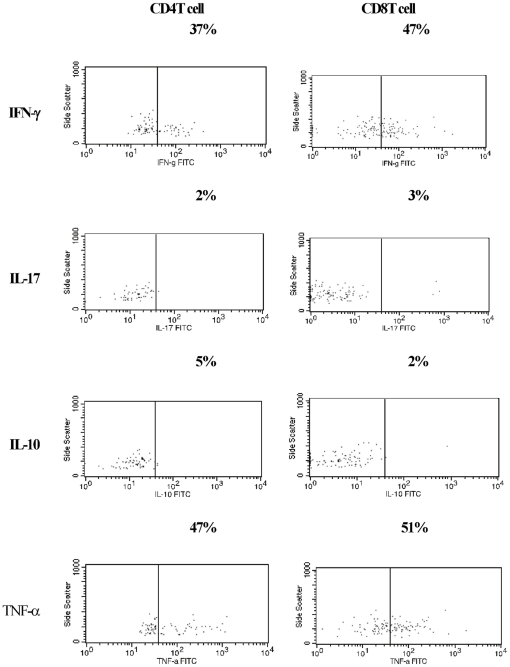
Characterization of the tumor infiltrating lymphocytes. One half of tumor infiltrating T cells from PMM2 tumor lesion were TNF-α producing cells. IFN-γ^+^CD4^+^ and IFN-γ^+^CD8^+^ T cells were also present in the tumor. By contrast, very few Th17 cells or Tr1 cells were detected in TILs. The number of infiltrating cells in the non-treated melanoma tumors was very limited, and thus was not analyzable. Representative results from five experiments are shown.

Single cell suspensions of TIL from PMM2 were analyzed. Abundant IFN-γ^+^CD8^+^ and IFN-γ^+^CD4^+^ cells were detected (Fig. 4). There was also an infiltration of TNF-α^+^CD4^+^ and TNF-α^+^CD8^+^ cells. On the other hand, Th17 and Tr1 cells were fewer in the TILs. The number of infiltrating cells was very limited in non-treated MM and could not be assessed.

## Discussion

In the present study, the effects of intra-tumor *P.acnes* vaccination (ITPV) on the growth of melanoma skin lesions was evaluated, and found that the growth of seeded melanoma cells was suppressed. ITPV successfully controlled melanoma progression *in vivo* by an induction of Th1 type cytokines, including TNF-α and IFN-γ in both the skin and the systemic circulation. The clinical benefit of vaccination was associated with subcutaneous granuloma formation. Tumor cells were not detected in the granulomas. The measured tumor size was significantly decreased in the vaccine-treated group compared with the control groups. The tumor size may have been underestimated because of the granuloma volume. However, granuloma formation is an immunological event that is related to augmented phagocytic activity as well as cellular cytotoxic activity. Granuloma formation thus plays an important role in effective anti-tumor immunotherapy. In the present study, we found that ITPV promotes the activation of TNF-α and IFN-γ producing cells in the skin tumor lesions. IL-12, TNF-α and IFN-γ are known to be effective anti-tumor cytokines. However, individual cytokines are reported to exert only limited clinical effects, and thus they have been most commonly used in combination with chemotherapy. Unlike the *in vitro* study results, the effect of recombinant cytokine therapy *in vivo* is limited, in large part due to the very short biological half-life of recombinant cytokines. A branched-chain polyethylene glycol moiety attached interferon alfa-2a (peginterferon alfa-2a) has been used to prolong the biological half-life [12]. Previous studies have shown that *P. acnes* enhances the anti-tumor activity of monocytes/macrophages [13], [14], [15] and the tumoricidal function of NKT and NK against melanoma [15]. In ITPV, *P. acnes* is phagocytosed and processed by monocytes/macrophages, which are present inside and around the tumors, and the persistent secretion of cytokines and chemokines from these cells leads to granuloma formation.

IL-12 is an antitumor cytokine that activates NK and cytotoxic T cells, thereby promoting strong anti-tumor activity by inducing IFN-γ [16], [17]. In the present study, we found increased local expression of IL-12p35 in ITPV. IL-12 is a heterodimeric cytokine containing IL-12p35 and IL-12p40 that binds to a specific receptor. On the other hand, free IL-12p40 forms sulfide-linked homodimers that block IL-12 function both *in vitro* and *in vivo* [18]. Enhanced expression of IL-12p35, but not IL-12p40, is suggested to have the potential to exert favorable therapeutic effects against tumors.

*P. acnes* binds to TLR2 on monocytes and dendritic cells, leading to activation of the IL-12 promoter [19]. IL-12 activates STAT-4 and T-bet transcription factors in T cells and NK cells. T-bet binds to the IFN-γ gene promoter and increases the production of IFN-γ [7]. In this study we found that IFN-γ induces cytotoxic effects by activating CD8^+^ T cells, NK cells and B cells. It also induces chemokines, including CXCL9 (MIG) and CXCL10 (IP-10) that suppress vascular proliferation.

Recombinant IL-12 has been used as an anti-cancer therapy, but with unsuccessful results, eliciting systemic side effects and only limited clinical benefit. Local administration of IL-12 for therapeutic purposes has been suggested to improve the outcome in certain cancers. To limit the expression of IL-12 and prolong local IL-12 secretion, IL-12 plasmid vaccination has been administered for metastatic melanomas [20]. In this regard, ITPV has the advantage of persistently inducing IL-12 expression at the site of injection; this is followed by infiltration of TNF-α and IFN-γ producing T cells into the lesions, resulting in tumor suppression.

Granuloma formation is a characteristic feature of *P. acnes* vaccination, with the accumulation of monocytes being required for this activity. Potent IFN-γ expression occurs after *P. acnes* administration and leads to granuloma formation. Granuloma is a persistent source of Th1 type cytokines *in vivo*. An increase in MIP2 (CXCL2) was detected in *P. acnes*-treated skin lesions. This chemokine is secreted by monocytes and macrophages, and is chemotactic for polymorphonuclear leukocytes and hematopoietic stem cells. MIP2 is one of the chemokines involved in granuloma formation. On the other hand, granuloma formation and ulceration have been considered as side effects in systemic anti-cancer vaccine trials. Since melanoma is a cutaneous malignancy, no special technique is required for accurate intralesional administration of the vaccine. Accumulated phagocytes in the granuloma may additionally contribute to the effective removal of tumor cells in combination with cytotoxic lymphocytes. Based on these observations, granuloma or ulceration is still considered to be relevant to successful cutaneous tumor immunotherapy.

In addition to its local effects, ITPV may also exert systemic anti-tumor activity. After ITPV, TNF-α and IFN-γ producing CD8^+^ T cells were increased in the spleen and skin. However, melanoma-specific cytotoxicity of CD8 T cells was not increased in the spleen or draining lymph nodes in *P. acnes* injected melanoma-bearing mice (Fig. S1), suggesting that most of the cytotoxic CD8 T cells may be recruited into the injected skin lesions. Consistent with the previous reports [21], [22], the number of splenic monocytes was also increased by ITPV. The role of the systemic immune response in the mechanism of distant metastasis remains unclear. Previous studies have suggested that augmentation of anti-tumor cytokine expression in spleen cells have preventive effects against distant metastasis.

In the present study, mice received either a single dose or two doses of vaccination. We injected *P. acnes* on day 0 and/or on day 14 into the cutaneous tumor lesions. Even the single therapy on day14 significantly suppressed the growth of melanoma cells. However, priming on day 0 followed by a second vaccination on day 14 resulted in a more potent growth inhibitory activity compared to the single vaccination. Early intervention before full tumor development may have the advantage of inducing enhanced Th1 type anti-tumor activity compared to vaccination after established melanoma growth. A second vaccination induced a booster effect on the activation of the cytokine network.

Th17, Tr1 and iTreg cells play critical roles in the regulation of the immune system. In the present study, *P. acnes* vaccination shifted the Th1/Th2 balance toward a dominant Th1 immune response. Th17 is involved in Th1-associated diseases such as psoriasis. However, we found no changes in the protein or RNA expression of IL-17 in the present immunotherapy, as IL-17 was undetectable in TIL. Therefore, it is unlikely that Th17 was involved in the beneficial effect of *P. acnes* vaccination therapy.

Tr1 and iTreg cells are known to regulate the inflammatory response. Tr1 regulatory cells produce IL-10 and play a critical role in the suppression of allergic diseases [11], [23]. It was reported that *P. acnes* therapy increases iTreg cells by stimulating TLR2 in Th2-mediated diseases [19], [24]. By contrast, suppression of iTreg cells has been associated with successful cancer immunotherapy [25]. Interestingly, neither Tr1 cells nor iTreg cells were elevated in the present study, suggesting that they are not involved in the mechanistic effect of this therapy. Unlike allergic mice, which have a Th2 dominant response, cancer-bearing mice may have different immunological backgrounds in response to *P. acnes* vaccination. Further investigation is required to clarify the precise mechanism of the *P. acnes* mediated immune responses.

In conclusion, the results of this study showed that ITPV successfully suppresses MM, and that the beneficial effect of this therapy depends on the induction of granuloma formation along with the secretion of IL-12, IFN-γ and TNF-α. Further investigation is required before this treatment comes into use in clinical practice. *P. acnes* vaccine is a promising candidate as an adjuvant therapy of melanoma.

## Supporting Information

Figure S1 **The cytotoxicity of CD8^+^ T cells prepared from spleen or draining lymph node was analyzed using three methods.** The first method is chromium release assay, and the second is viability detection by flow cytometry using Live/Dead cell-mediated cytotoxicity kit (Molecular probes, Carlsbad, CA). Finally DHL cell cytotoxicity assay kit (AnaSpec Corporate Headquarter, San Jose, CA) was used to detect the release of Lactate Dehydrogenase (LDH) from targeted melanoma cells. Spleen and draining lymph node samples were taken from melanoma and *P. acnes*-injected mice: melanoma cell was free in the dorsal skin, *P. acnes* only injected mice, and normal control mice. Single cell suspensions were prepared by mechanical mincing, and after passing through a 70-µm-pore mesh, the cells are washed and resuspended in PBS. After Ficoll separation, the cells were washed and resuspended in RPMI1640 medium containing 10% FBS. CD8 T cells were purified using magnetic beads, and co-cultured with B16 melanoma cells at three different effector cell/target cell ratio (12.5∶1, 25∶1, 50∶1) according to previous reports. Chromium release assay **A,** 6 hours incubation LN CD8 T cells. **B,** 6 hours incubation splenic CD8 T cells. **C,** 15 hours incubation LN CD8 T cells. **D,** 15 hours incubation splenic CD8 T cells. When there was injury of targeted melanoma cells, chromium was released. Analysis of apoptotic melanocytes using live/dead viability detection system by flow cytometry **E,** 8 hours incubation LN CD8 T cells. **F,** 8 hours incubation splenic CD8 T cells. Analysis of lactate dehydrogenase (LDH) released from targeted melanoma cells **G,** 8 hours incubation LN CD8 T cells **H,** 8 hours incubation splenic CD8 T cells. Melanoma and *P. acnes*-injected mice: •, *P. acnes* only injected mice: ▪, and normal control mice: ▴. CD8 T cells melanoma-specific cytotoxicity was not increased in *P. acnes* injected melanoma-bearing mice, suggesting that most cytotoxic CD8 T cells was recruited into the injected skin lesions.
(TIFF)
