# Supplementary figures and images for: Intratumoral Injection of Propionibacterium acnes Suppresses Malignant Melanoma by Enhancing Th1 Immune Responses
Source: PLoS One. 2011 Dec 21;6(12):e29020. doi: 10.1371/journal.pone.0029020 (PMC3244427; doi:10.1371/journal.pone.0029020)

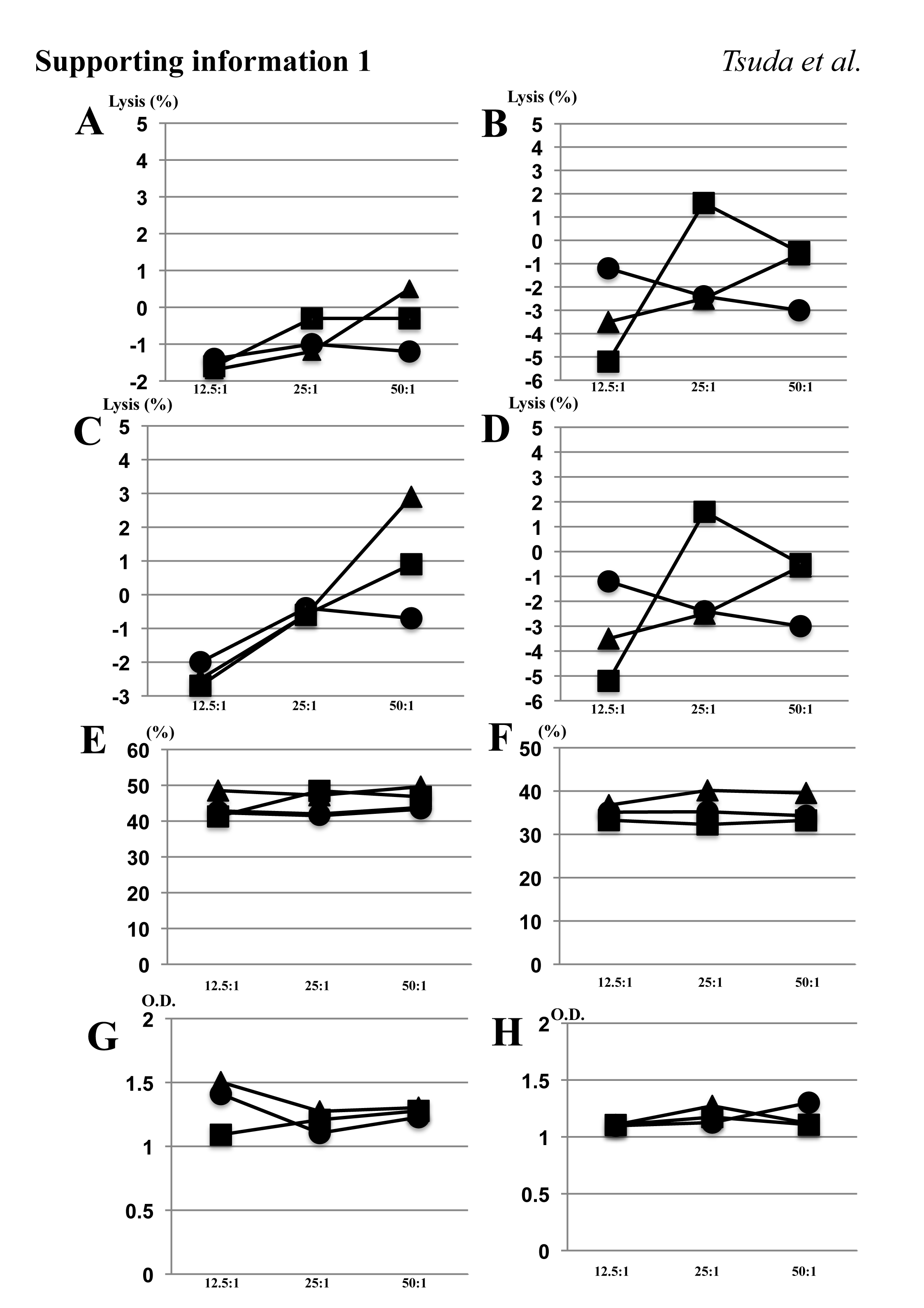

Supplement: Figure S1 — The cytotoxicity of CD8+ T cells prepared from spleen or draining lymph node was analyzed using three methods. The first method is chromium release assay, and the second is viability detection by flow cytometry using Live/Dead cell-mediated cytotoxicity kit (Molecular probes, Carlsbad, CA). Finally DHL cell cytotoxicity assay kit (AnaSpec Corporate Headquarter, San Jose, CA) was used to detect the release of Lactate Dehydrogenase (LDH) from targeted melanoma cells. Spleen and draining lymph node samples were taken from melanoma and P. acnes-injected mice: melanoma cell was free in the dorsal skin, P. acnes only injected mice, and normal control mice. Single cell suspensions were prepared by mechanical mincing, and after passing through a 70-µm-pore mesh, the cells are washed and resuspended in PBS. After Ficoll separation, the cells were washed and resuspended in RPMI1640 medium containing 10% FBS. CD8 T cells were purified using magnetic beads, and co-cultured with B16 melanoma cells at three different effector cell/target cell ratio (12.5∶1, 25∶1, 50∶1) according to previous reports. Chromium release assay A, 6 hours incubation LN CD8 T cells. B, 6 hours incubation splenic CD8 T cells. C, 15 hours incubation LN CD8 T cells. D, 15 hours incubation splenic CD8 T cells. When there was injury of targeted melanoma cells, chromium was released. Analysis of apoptotic melanocytes using live/dead viability detection system by flow cytometry E, 8 hours incubation LN CD8 T cells. F, 8 hours incubation splenic CD8 T cells. Analysis of lactate dehydrogenase (LDH) released from targeted melanoma cells G, 8 hours incubation LN CD8 T cells H, 8 hours incubation splenic CD8 T cells. Melanoma and P. acnes-injected mice: •, P. acnes only injected mice: ▪, and normal control mice: ▴. CD8 T cells melanoma-specific cytotoxicity was not increased in P. acnes injected melanoma-bearing mice, suggesting that most cytotoxic CD8 T cells was recruited into the injected skin lesions. (TIFF) [file pone.0029020.s001.tiff]
